# Analgesia Effect of Enteric Sustained-Release Tetrodotoxin Pellets in the Rat

**DOI:** 10.3390/pharmaceutics12010032

**Published:** 2020-01-01

**Authors:** Bihong Hong, Jianlin He, Jipeng Sun, Qingqing Le, Kaikai Bai, Yanhua Mou, Yiping Zhang, Weizhu Chen, Wenwen Huang

**Affiliations:** 1 Third Institute of Oceanography, Ministry of Natural Resources, Xiamen 361005, China; 2 Technical Innovation Center for Utilization of Marine Biological Resources, Ministry of Natural Resources, Xiamen 361005, China; 3 Department of Pharmacology, Shenyang Pharmaceutical University, Shenyang 110016, China

**Keywords:** tetrodotoxin, pharmacokinetic study, sustained-release, enteric pellets, analgesia

## Abstract

Tetrodotoxin (TTX) was identified as a latent neurotoxin that has a significant analgesia effect. It was rapidly absorbed and excreted in rat after intramuscular (i.m.) injection. To maintain the effect, frequent injections were required. The enteric sustained-release TTX pellets with sucrose pellets as a drug carrier was prepared by fluidized bed spray irrigation, coated in sequence with Eudragit NE30D as a sustained-release layer, hydroxypropyl methylcellulose (HPMC) as a barrier layer and Eudragit L30D-55 as an enteric coating. TTX in the pellets could be sustained released for 12 h in dissolution test. In vivo, TTX pellets reached *C*_max_ at 5 h, and *t*_1/2_ was 14.52 ± 2.37 h after intragastrically (i.g.) administration in rat. In acetic acid induced writhing test in rat, the pellets at the dosages of 20, 40, 60 and 80 μg·kg^−1^ produced analgesic effect at about 1.5 h to 9 h and the strongest effect was at about 3 h to 6 h. Simultaneously, the LD_50_ of the enteric sustained-release TTX pellets was 840.13 μg·kg^−1^, and the ED50 was about 30 μg·kg^−1^. Thus, the therapeutic index was about 25. The enteric sustained-release TTX pellets with absolute analgesia effect and greatly enhanced safety was prepared.

## 1. Introduction

Tetrodotoxin (TTX), found in puffer fish and other terrestrial animals, was identified as a latent neurotoxin. TTX blocks the voltage-gated sodium channels (VGSCs) in nerves with high selectivity [1]. The VGSCs can be classified as TTX-sensitive (TTX-S) and TTX-resistant (TTX-R) subtypes, characterized in terms of their sensitivity to TTX [2]. It has been extensively used to clarify the role of VGSCs subtypes in the wide range of physiological and pathophysiological processes in the nervous system [3].

TTX has a significant analgesia effect on cancer pain, acute pain, inflammation pain, neuralgia and also the pain caused by arthritis, trauma, burn, and contusion. There is no drug tolerance and addiction reported in all of those TTX therapies [2]. The mechanism of analgesia may be due to blockage of the VGSCs, leading to decrease of excitatory conduction and nerve impulse of nerve cells at the injury sites, and even influence on plasticity of the nervous system.

Since TTX is unstable in gastric acid, the drug was administrated via intramuscular (i.m.) or subcutaneous (s.c.) injection in most studies [4]. However, both the activity and toxicity of TTX is high. Thus, the therapeutic window of TTX is narrow, especially with i.m. or s.c. injections. Considering the safety of systemic administration, quantity control of the effective content of the preparation is also a challenge. Further, the half-life (*t*_1/2_) of TTX is short, and the effective blood concentration can only be maintained for about 2 h after i.m. injection [5]. In order to maintain the efficacy, frequent i.m. administration may be required, leading to poor clinical compliance. Therefore, there is an obvious need to develop a formulation that can expand the therapeutic window and improve the clinical compliance to promote the clinical application of TTX.

A single dose of TTX is usually less than 300 μg in humans, therefore, it is difficult to control the content uniformity, which is a crucial restriction of oral preparations. Pellets are defined as spherical and free-flowing granules, with narrow size distribution varying between 0.5–1.5 mm [6]. Pellets are most often used to reduce the gastric irritation effect and increase gastrointestinal transit times for decreasing gastric emptying effect [7]. The flexible pellets blend could lower the risk of dose dumping [8]. The insoluble polymer layer covering the pellets masks any undesirable taste of the drug and improves drug stability [9]. In addition, the coating imperfection of a small portion of the pellets would have little influence on drug release, because each pellet acts as a single drug reservoir [6]. With the development of the preparation techniques of pellets, such as the sustained-release and controlled-release techniques, the content validity and safety of the pharmaceutical has been improved, therefore, pellets have drawn extensive attention on novel drug development.

Extrusion-spheronization (Ex-Sp) and fluid-bed processing are the main commercialized methods for pellets preparation [10]. The Ex-Sp has advantages of high-speed preparation, high loading and content uniformity, and it is mainly used to prepare the drug-loaded pellets and sugar sphere [11]. The fluid-bed processor can perform multiple tasks like coating, drying, granulation and pelletizing with high efficiency. In addition, it protects product against moisture, light and air. The powder recycles equipment at the bottom and can prevent the waste of raw material and recycle organic solution to improve the working environment and reduce the cost. In addition, fluid-bed processing embodies advantages like high drug loading and content uniformity. It is specially suitable for the loading of low content drugs [12,13].

In this study, based on the advantages of pellets [14], enteric sustained-release TTX pellets were prepared via fluid-bed processing, to overcome the disadvantages of the TTX injection administrations, like short *t*_1/2_ and poor compliance. A writhing test was performed to evaluate the analgesia effect of the TTX pellets. The pharmacokinetic profile of TTX pellets was evaluated in vivo and in vitro. In addition, the acute toxicity study of the pellets was performed in rats.

## 2. Materials and Methods

### 2.1. Materials

Tetrodotoxin (purity≥98%) was provided by ZhaoYang Biopharma Co., Ltd. (Xiamen, China). Sugar sphere (600–710 μm) and microcrystalline cellulose sphere (500–710 μm) were purchased from JRS Pharma Co., Ltd. (Rosenberg, Germany). Opadry (YS-1-7006) was purchased from Colorcon Co., Ltd. (Shanghai, China). Hydroxypropyl methylcellulose (E5) was purchased from Huzhou Zhanwang Pharmaceutical Co., Ltd. (Huzhou, China). 1-octanesulfonate sodium was provided by REGIS Technologies (Morton Grove, IL, USA). Sodium phosphate monobasic dehydrate, sodium phosphate dibasic dehydrate, sodium hydroxide, phosphoric acid and acetonitrile were provided by Sinopharm Chemical Reagen Co., Ltd. (Shanghai, China). Triethyl citrate was provided by Shanghai Macklin Biochemical Co., Ltd. (Shanghai, China). Talc (10 μm) was purchased by Guangxi Longsheng Huamei Talc Development Co., Ltd. (Guilin, China). Citric acid was purchased from Taishan Xinning Pharmaceutical Co., Ltd. (Taishan, China). Poly (1-carboxyethylene) (Eudragit NE 30D, RS 30D, RL 30D, L30D-55) was purchased from Evonik Industries AG (Darmstadt, Germany).

### 2.2. Animals

Experiments were carried out on adult Sprague–Dawley (SD) rats (180–220 g) purchased from the Experimental Animal Center of Shenyang Pharmaceutical University. All procedures were approved by the Animal Care Committee at Shenyang Pharmaceutical University (ethical committee approval numbers: SYPU-IACUC-C2017-11-22-201, Date (22/11/2017) and SYPU-IACUC-C2017-12-15-208, Date (15/12/2017)). The rats were acclimatized to the laboratory for at least 3 days before experiments. The rats were housed at five per cage with a 12-h light/dark cycle under controlled temperature (20–24 °C) and humidity (45–65%). Food and water were allowed ad libitum during the study period.

### 2.3. Methods

#### 2.3.1. HPLC Conditions for Studies In Vitro

HPLC separation was performed on Waters 2695 (Waters, Milford, MA, USA) by using a ZORBAX SB-C8 column (4.6 mm × 250 mm, 5 μm). The mobile phase comprised of 1-octanesulfonate sodium-phosphate buffer (8.95 g sodium phosphate dibasic dehydrate, 3.90 g sodium phosphate monobasic dehydrate, 0.27 g 1-octanesulfonate sodium were dissolved in distilled water up to 500 mL, with stirring and filtrating) with the flow rate of 0.3 mL·min^−1^ and the temperature was maintained at 28 °C. In this experiment, HPLC combined with a fluorescence detector (Waters 2475, Milford, MA, USA), having intensity monitored at 365 nm and 510 nm, excitation and emission wavelengths, respectively. The post column reaction reagent was 4 mol·L^−1^ NaOH, and the injection volume was 10 μL at a flow rate of 0.3 mL·min^−1^ at the temperature of 110 °C.

#### 2.3.2. The Stability of Tetrodotoxin (TTX) in Dissolution Medium

TTX standard solution was prepared by diluting 1 mg TTX in 10 mL 0.0035% (*w*/*w*) citric acid. A 1 mL standard solution sample was diluted in distilled water, 0.1 mol·L^−1^ HCl, or phosphate buffer solutions (pH 5.8 and pH 6.8) to 10 mL, respectively. The solutions were placed in water bath at 37 °C under agitation for 12 h. Subsequently, the samples were withdrawn at 0 h, 8 h and 12 h that the concentrations of TTX could be calculated from the peak heights of HPLC using the corresponding linear regression equation in an external standard calibration method.

#### 2.3.3. TTX Pellets Release In Vitro

The dissolution test instruments (RC806D, TDTF, Tianjin, China) was employed in dissolution test for pellets based on the paddle method in Chinese Pharmacopeia 2010. The enteric sustained-release TTX pellets were placed in water bath at 37 ± 0.5 °C, and the paddle speed was at 100 r·min^−1^. In order to simulate environment in human gastrointestinal tract, the dissolution medium was 0.1 mol·L^−1^ HCl (100 mL) at the initial 2 h. Then the sample was withdrawn, and HCl solvent was removed. Simultaneously, 100 mL phosphate buffer solution (pH 5.8) acted as dissolution medium. An amount of 2 mL of each sample was withdrawn from the vessel at predetermined time intervals, and replaced with 2 mL of fresh medium. The withdrawn samples were filtered through a 0.45-μm membrane and analyzed with HPLC system. The TTX concentrations could be calculated from the peak heights of HPLC using the corresponding linear regression equation in an external standard calibration method.

### 2.4. Preparation of Enteric Sustained-Release TTX Pellets

The pelletizing process was carried out in a fluid bed rotor (WBF-5G, Chongqing Enger Granulating & Coating Technological, Chongqing, China). The enteric sustained-release pellets were composed of drugs-loaded pellets, a sustained-release layer, a barrier layer and an enteric layer for adjusting the kinetics of drug release.

#### 2.4.1. Preparation of Drug-Loaded TTX Pellets

Working solution of 0.4 mg/mL TTX was prepared by diluting TTX in 0.1% citric acid (*w*/*w*) with 0.5% hydroxypropyl methylcellulose (HPMC) (*w*/*w*) as the binder. The pellets coating was performed using a fluid bed of the bottom spray type [15]. Pellets of 400 g were used for drug-loaded operation with the experimental conditions as follows: air velocity of 150 m^3^·h^−1^, pump rate of 2 r·min^−1^, inlet temperature of 40 °C, solution temperature of 25–30 °C, spray pressure of 0.16 MPa. Finally, pellets were dried at 40 °C in the fluid bed for 15 min.

#### 2.4.2. Preparation of Sustained-Release Layer TTX Pellets

The drug-loaded pellets were transferred into a fluid bed. The coating suspension was prepared as follows: talc powder (100% of the dry polymer weight) was homogenized with a blend of water for 10 min. Then it was added to the Eudragit NE30D aqueous dispersions under agitation to obtain a mixture comprised of 12.5% polymer. The mixture was passed through a 250 µm sieve and continuously stirred. The coating suspension was agitated during the coating process to maintain homogeneity in the formulation. The coatings operation conditions used in this study were as follows: air velocity of 180 m^3^·h^−1^, pump rate of 5 r·min^−1^, inlet temperature of 35 °C, material temperature of 25–30 °C, spray pressure of 0.3 MPa. Finally, pellets were dried at 40 °C in fluid bed for 15 min.

#### 2.4.3. Preparation of Barrier Layer TTX Pellets

The pellets containing sustained-release film were transferred into a fluid bed. Talc (50% of the dry polymer weight) was mixed in 2.5% (*w*/*w*) HPMC solution for 10 min in a homogenizer. The coating mixture was filtered through a screened mesh size of 250 µm diameter. The coating suspension was agitated during the coating process. The coatings operation conditions used in this study were as follows: air velocity of 180 m^3^·h^−1^, pump rate of 7 r·min^−1^, inlet temperature of 40 °C, material temperature of 25–35 °C, spray pressure of 0.3 MPa. Finally, pellets were dried at 40 °C in a fluid bed for 15 min.

#### 2.4.4. Preparation of Enteric Layer TTX Pellets

The pellets containing barrier layer were transferred into a fluid bed. The coating suspension was prepared as follows: triethyl citrate (10% of the dry polymer weight), and talc powder (25% of the dry polymer weight) was homogenized with a blend of water for 10 min, then added to the Eudragit L30D-55 aqueous dispersions under agitation to obtain a mixture comprised of 12.5% polymer. The mixture was passed through a 250 µm sieve and continuously stirred. The coating suspension was agitated during the coating process. The coatings operation conditions used in this study were as follows: air velocity of 180 m^3^·h^−1^, pump rate of 7 r·min^−1^, inlet temperature of 40 °C, material temperature of 30–35 °C, spray pressure of 0.3 MPa. Finally, pellets were dried at 40 °C in a fluid bed for 15 min.

#### 2.4.5. The Optimization and Repetitiveness of Enteric Sustained-Release TTX Pellets

To confirm the optimized preparation process and investigate the release repeatability of the enteric sustained-release TTX pellets, the dissolution test of 3 consecutive batches enteric sustained-release pellets was performed with the paddle method.

### 2.5. Acetic Acid-Induced Writhing Test

Male rats were placed in a clear acrylic chamber (20 × 20 × 30 cm) to acclimatize the test conditions for 30 min. Saline, TTX formulation (20, 40, 60 and 80 μg/kg) or ibuprofen (100 mg/kg) was intragastrically (i.g.) administrated at 0 h. Then, 0.6% acetic acid solution (10 mL/kg, intraperitoneally) was administrated at different time points (1.5 h, 3 h, 6 h, 9 h, 12 h, 18 h and 24 h). The contractions of the abdomen, elongation of the body, twisting of the trunk and/or pelvis ending with the extension of the limbs were considered as complete writhing. At each time point, the number of writhing during a 30-min duration following acetic acid solution injection was counted and recorded. Results were expressed as mean percent inhibition of writhing: inhibition (%) = [(mean writhing numbers of control − mean writhing numbers of treatment)/mean writhing numbers of control] × 100%.

### 2.6. An Acute Oral Toxicity Study

A total of 50 SD rats were randomly divided equally in 5 groups (half male and half female). Before the administration of different doses of TTX formulation, the rats were fasted for 12 h with water ad libitum. The 5 groups of rats were given 1466, 1100, 825, 618, and 464 μg/kg body weight of the sustained-release TTX pellets with a single oral administration, respectively. Then, the animals were continuously observed during the first 1 h for the general behavior and signs of toxicity, then intermittently observed for 4 h, and thereafter over a period of 24 h. The animals were observed daily in 7 consecutive days. The median lethal dose (LD_50_) and the probit-log(dose) equations were calculated by way of the bliss method with a BD-6240 Biological Data Acquisition and Analysis System (Chengdu Technology & Market Corp., Ltd., Chengdu, China).

### 2.7. Pharmacokinetics Studies

#### 2.7.1. Administration and Blood Samples

For the sustained-release TTX pellets, a group of 6 rats (260 ± 20 g), half male and half female, with jugular vein catheterization (JVC), were administrated i.g. with a single dose of 150 μg/kg. Serial blood samples were collected in heparinized tubes via the jugular vein before and at time points of 1, 2, 3, 4, 5, 6, 8, 10, 18, 21, 24, 32, and 48 h after administration. About 0.2 mL blood was collected before and at each time point of 1, 2, 3, 4, 5, 6, 8, and 10 h, and about 0.3 mL blood was collected at each other time point (Figure S1). Plasma was separated and stored frozen at −20 °C until analysis.

For intravascular (i.v.) injection of TTX, a group of 6 rats (260 ± 20 g), half male and half female, with JVC, were treated with a single dose of 6 μg/kg. Serial blood samples were collected in heparinized tubes via the jugular vein before and at time points of 0.08, 0.17, 0.25, 0.5, 0.75, 1.0, 1.25, 1.5, 2, 4, 6, 8, and 12 h after administration. About 0.2 mL blood was collected before and at each time point of 0.08, 0.17, 0.25, 0.5, 0.75, 1.0, 1.25, 1.5 and 2 h, and about 0.3 mL blood was collected at each other time point (Figure S1). Plasma was separated and stored frozen at −20 °C until analysis.

#### 2.7.2. Pharmacokinetic Analysis

A pharmacokinetic analysis using the DAS 2.0 software was performed to determine the key parameters including the maximum plasma concentration (*C*_max_), the time to maximum concentration (*T*_max_), *t*_1/2_, the area under the plasma concentration-time curve from zero to the end time point (AUC_0–t_), and the area under the plasma concentration-time curve from zero to infinity (AUC_0–∞_). The oral bioavailability (*F*) is measured by comparing AUC value according to the following equation:

*F* = (AUC_i.g._/Dose_i.g._)/(AUC_i.v._/Dose_i.v._) × 100%

### 2.8. Statistial Analysis

Data were presented as mean ± SD. Statistical analysis were performed with one-way ANOVA followed by Turkey’s multiple comparison test. Significant differences were considered when *p* value was less than 0.05.

## 3. Results

### 3.1. Stability of TTX in Dissolution Medium

As shown in Table 1, the stability of TTX in 4 dissolution mediums was investigated. TTX was stable in pure water and pH 5.8 sodium-phosphate buffer, relatively stable in 0.1 mol·L^−1^ HCl solution. However, TTX was easily decomposed in pH 6.8 buffer, which could influence the accuracy of release test. Therefore, the release of TTX pellets in vitro was investigated in pH 5.8 sodium-phosphate buffer.

### 3.2. Effect of Sustained-Release Layer Weight on TTX Pellets Release

**Figure 1:**
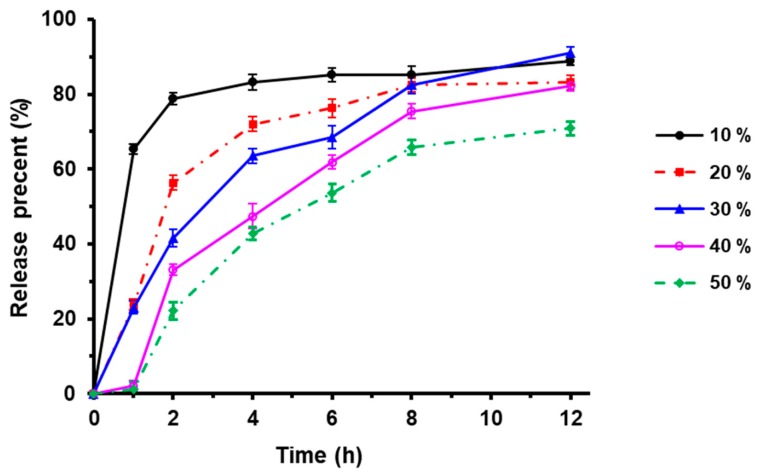
Effect of sustained-release coating weight on drug release (*n* = 3).

Eudragit NE 30D is a neutral copolymer composed of ethyl acrylate and methyl methacrylate with a ratio of 2:1, which could be used to prepare sustained-release drugs. As shown in Figure 1, 80% TTX was released in the preparations containing different weight of the sustained coating Eudragit NE 30D (10%, 20%, 30%, 40% and 50% of the pellets) in the dissolution medium in 12 h. Further, TTX could be released at a consistent rate for 12 h when the weight of sustained coating was 40% of the pellets.

### 3.3. Effect of Barrier Layer Weight on Drug Release

Since Eudragit L30D-55 which is acidic would be used as the enteric layer in the next step, it is necessary to have a barrier layer to prevent the reaction between the sustained layer and the enteric layer during preparation and storage.

**Figure 2:**
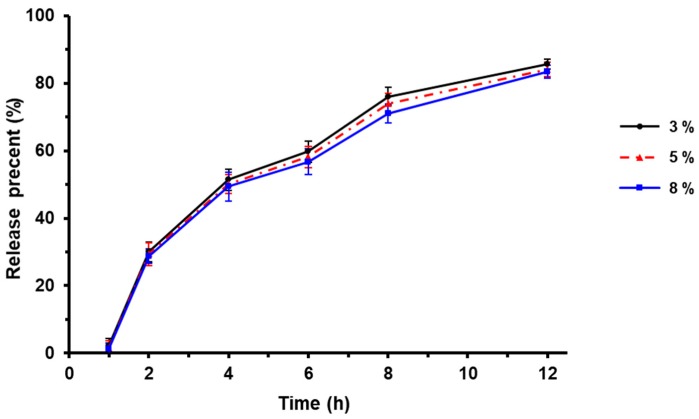
Effect of barrier layer weight on drug release (*n* = 3).

In this study, HPMC was used as the barrier layer. The influence of HPMC weight on TTX release was investigated by the weight of 3%, 5% and 8% of the pellets. As shown in Figure 2, there was no significant effect of barrier layer weight on TTX release in the dissolution medium. In this study, HPMC of 5% weight was chosen as the barrier layer.

### 3.4. Effect of Enteric Layer Weight on Drug Release of TTX Pellets

Eudragit L30D-55 is a copolymer composed of methacrylic acid and ethyl acrylate with a ratio of 1:1, which was used as the enteric layer of the TTX pellets.

**Figure 3:**
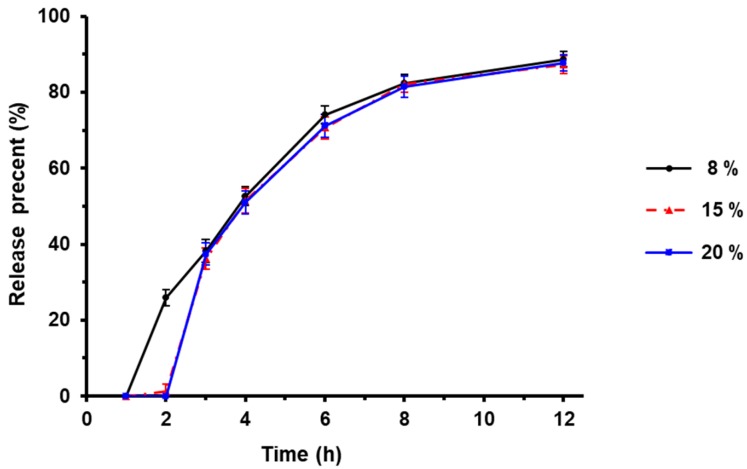
Effect of enteric layer weight on drug release (*n* = 3).

The influence of enteric layer weight on TTX release in vitro was investigated by 8%, 15% and 20% of the pellets in the dissolution medium. As shown in Figure 3, when the enteric layer weight was 8%, the drug release of enteric pellet was greater than 10% in the first 2 h when in acid solution. We observed that the pellets appeared adhesion in acid solution when enteric layer weight was 8% or 15%, which indicated poor acid resistance. Therefore, 20% of enteric layer weight was chosen for the following research.

### 3.5. The Repeatability of Enteric Sustained-Release TTX Pellets Release In Vitro

**Figure 4:**
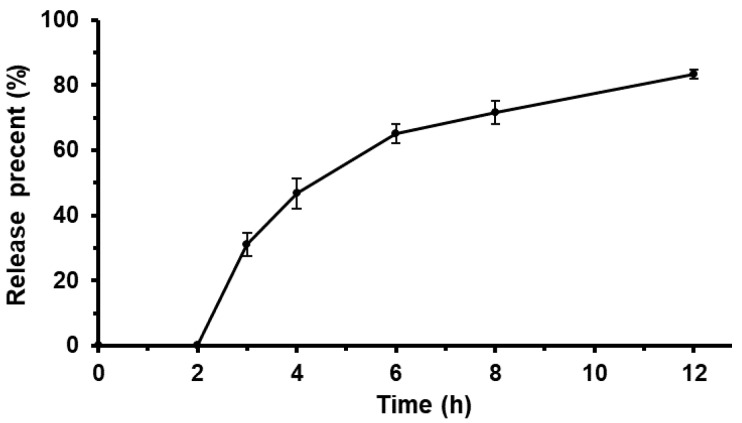
Effect of enteric sustained-release pellets on drug release (*n* = 3).

According to the preparation process of the pellets, the drug release in vitro was investigated in 0.1 mol·L^−1^ HCl solution for the first 2 h, and then in pH 5.8 sodium-phosphate buffer for 2–12 h. The drug release with three batches of enteric sustained-release pellets were shown in Figure 4.

For all the threee batches, 0% of TTX was released at the first 2 h, but greater than 80% was released at 2–12 h. The samples were sustained and released without a burst release at 2–12 h. The results indicated that the experiments of enteric sustained-release pellets released in vitro could be well repeated.

### 3.6. Effects of Sustained-Release TTX Pellets against Acetic Acid-Induced Pain

**Figure 5:**
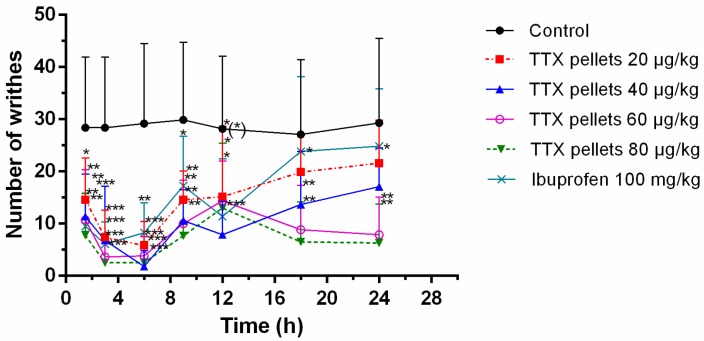
Number of the writhes of acetic acid treated rats after sustained-release TTX pellets treatment. Saline, TTX formulation or ibuprofen was intragastrically (i.g.) administrated at 0 h. Then, 0.6% acetic acid solution (10 ml/kg, intraperitoneally) was administrated at different time points (1.5 h, 3 h, 6 h, 9 h, 12 h, 18 h and 24 h). At each time point, the number of writhing during a 30-min duration following acetic acid solution injection was counted and recorded. Data are expressed as mean ± SD (*n* = 10). * *p* < 0.05, ** *p* < 0.01 and *** *p* < 0.001 compared with control.

A writhing test was used to evaluate the antinociceptive effect of TTX pellets (Figure 5). The pellets initiated their effects at about 1.5 h after oral administration. Particularly, 40 μg/kg TTX pellet had prominent efficacy among all tested doses at 12 h. The peak effect of 20 μg/kg, 60 μg/kg and 80 μg/kg appeared at 3 h or 6 h, especially 80 μg/kg showed the best efficacy among all doses at 1.5–6 h. The efficacy diminished from 9 h to 12 h except for 60 μg/kg and 80 μg/kg, which even had increased efficacy from 12 h. At 18–24 h, it appeared that 20 μg/kg still had sustained efficacy though it had no significance compare with control, while 60 μg/kg had an increased efficacy from 18 h to 24 h, also 80 μg/kg had an increase from 12 h to 24 h. Ibuprofen showed an effect similar to TTX pellets of 20 μg/kg. Though at 12 h it still had some effects, it had no significance. However, TTX pellets of 40 μg/kg, 60 μg/kg and 80 μg/kg sustained their efficacy during the whole experiment duration, indicating their longer duration compared to ibuprofen.

**Figure 6:**
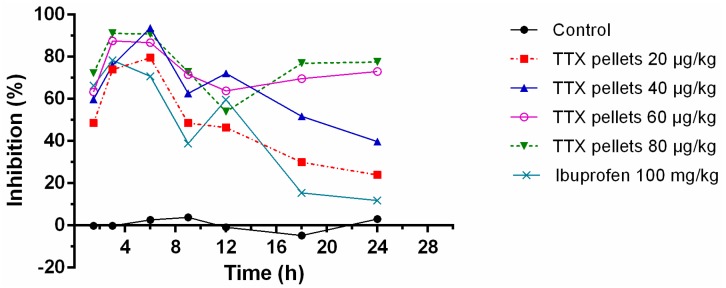
The percentage of inhibition of sustained-release TTX pellets on writhing test. Saline, TTX formulation or ibuprofen was i.g. administrated at 0 h. Then, 0.6% acetic acid solution (10 mL/kg, intraperitoneally) was administrated at different time points (1.5 h, 3 h, 6 h, 9 h, 12 h, 18 h and 24 h). At each time point, the number of writhing during a 30-min duration following acetic acid solution injection was counted and recorded. Data are expressed as mean (*n* = 10).

The percentage of inhibition of sustained-release TTX pellets at most time points could obtain 50% or above in a dose-dependent manner (Figure 6). The maximum effect appeared at 3–6 h. The inhibitory effect declined by time. However, for the 60 μg/kg and 80 μg/kg, their inhibitory effect could rise up again at 9–12 h, indicating their higher potential of antinociceptive effects with longer duration.

### 3.7. The Acute Toxicity of Oral Sustained-Release TTX Pellets

To evaluate the safety of enteric sustained-release TTX pellets, the acute toxicity test was performed in half female and half male SD rats at the doses from 464 μg/kg to 1466 μg/kg body weight.

There was no obvious intoxication reaction when rats were treated with sustained-release TTX pellets at low dose. At 2 h after administration, poisoning symptoms like lack of movement, wheezing, eyelid weakness, and muscle fibrillation gradually appeared in high dose rats, and death numerously occurred at 3–8 h, until 20 h. There was no obvious change in the gross anatomy of the dead animals. The death in the acute toxicity test was shown in Table 2. Based on the bliss method, the LD_50_ was 840.13 μg·kg^−1^ and the 95% confidence interval was 706.32~999.28 μg·kg^−1^. The LD_50_ of female and male rats were 771.65 μg·kg^−1^ and 927.98 μg·kg^−1^, respectively.

The LD_50_ regression equation of sustained-release TTX pellets for oral administration is *Y* = −13.4052 + 6.2938 × log(*D*).

The animals’ body weights after 7 days are shown in Table 3. The distribution of animal weight before administration of the drug was quite average. No significant diversity was found between the rats’ increasing weight during the experiment duration.

Taken together, sustained-release TTX pellets (40, 60, 80 μg/kg) used for acute pain-induced by acetic acid, showed a good antinociceptive effect within 3–9 h and lasted for more than 24 h. Moreover, the LD_50_ of sustained-release TTX pellets was 840.13 μg/kg, which is much higher than the effective dose in writhing test.

### 3.8. Pharmacokinetic Studies of Sustained-Release TTX Pellets

#### 3.8.1. Studies of Intravenous (i.v.) TTX Administration

**Figure 7:**
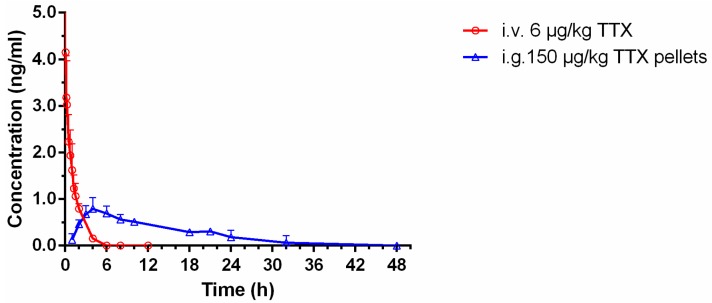
Mean plasma concentration-time profiles of TTX in rats following i.v. administration of TTX injection at 6 μg/kg body wt. (red curve) and i.g. administration of sustained-release TTX pellets at 150 μg/kg body wt. (blue curve) (*n* = 6, mean ± SD).

The time-courses of TTX concentrations in the blood after i.v. injection at a dose of 6 μg/kg to rats were shown in Figure 7 (red curve). The blood concentration of TTX was 4.12 ng/mL at 0.08 h after administration, then it plunged within 10 min, and declined gradually. The pharmacokinetic parameters of TTX with i.v. injection are summarized in Table 4.

#### 3.8.2. Studies of i.g. Sustained-Release TTX Pellets Administration

As shown in Figure 7 (blue curve) and Table 5, the blood concentration of sustained-release TTX pellets reached 0.88 ng/mL at 5 h. Then it declined gradually until 48 h.

The AUC_0–t_ of i.g. administration of the TTX pellets was 10.77 ± 2.28 ng·h/mL, and AUC_0–∞_ was 16.76 ± 2.93 ng·h/mL. Meanwhile, the *t*_1/2_ of i.g. administration was 14.52 ± 2.37 h, which was much higher than that of i.v. administration (0.92 ± 0.17 h), indicating a long-lasting effect of sustained-release pellets administration. The *C*_max_ was 0.88 ± 0.22 ng/mL, which was much lower than that of the i.v. administration (4.12 ng/mL). The value of *V*_d_ was 192,752.13 ± 46,492.16 mL/kg, suggesting a low plasma concentrations of TTX, and TTX might mainly distributed in tissue or be confined in a certain organ, properly in intestine according to our previous study [5]. Compared with the results of i.v. administration of TTX (considered as 100%), the oral bioavailability of sustained-release TTX pellets was 9.7% (Table 5).

## 4. Discussion

It’s been reported that TTX has an analgesic effect. The analgesic effect of TTX can last for weeks or even months in human, which is thought to be due to blockage of sodium channels, especially Na_v_1.7 [17]. In a multicenter open-label longitudinal trial, 17 of 31 i.m. TTX treatments resulted in clinical meaningful reduction of severe, treatment resistant cancer pain, and the relief of pain persisted for two weeks or longer [13]. The research also showed that i.m. TTX is generally safe and well tolerated. Additionally, TTX did not cause drowsiness like morphine in reducing peripheral and central pain. In our study, the analgesic effect of single dose of TTX pellets also last for a longer time than expected.

TTX effects on neuropathic pain were widely investigated, since TTX-S VGSCs are crucial players for neuropathic pain [2]. Low doses of TTX can be useful to prevent and treat paclitaxel-induced neuropathic pain, and TTX-S subtypes of sodium channels also play a role in the pathogenesis of chemotherapy-induced neuropathic pain [18]. Low dose of TTX locally applied in dorsal root ganglion neuron could inhibit the neuropathic pain caused by the spinal nerve ligation without blocking action potential conduction [19]. TTX was reported to block the conduction of the sciatic nerve in mice to inhibit the thermal and mechanical pain [14]. In rat chronic constrictive injury and spared nerve injury neuropathic pain models, effective pre-emptive analgesia can be achieved only when TTX is administered early after injury and the effect lasted for several days [14]. Administration of TTX relieved chronic constriction injury-induced behavioral hypersensitivity via attenuating activation of astrocytes in the cuneiform nucleus [20].

The inflammation induced by the allergy, thermal stimulation and mechanical pain could be inhibited by TTX [21]. The inflammatory pain of rats could be reduced with the systemic administration [22]. Subcutaneous injections of TTX decreased pain behavior in the formalin test at the 6 μg·kg^−1^ and in the writhing test at 3 and 6 μg·kg^−1^ without any motor deficit, respiratory distress or sedation [12]. TTX diminished mechanical allodynia and thermal hyperalgesia with an ED_50_ of 1.08 (0.89) and 0.62 (0.33) μg·kg^−1^, respectively. The research stated that TTX showed a dose-dependent effect on decreasing inflammation [12]. Inflammation by formalin occurred in the second stage after acute pain [23], and TTX slightly decreased acute pain but more effectively inhibited second stage inflammation by formalin [12]. Besides the effect of TTX on neuron cells, it was proposed that sodium channel blockers like TTX might also have effect on immune cells to attenuate inflammatory pain [24]. Carbamazepine is another voltage-gated sodium channel blocker. The relative antinociceptive potencies of intrathecal carbamazepine versus TTX were approximately 1:300 in chronic inflammatory rats [25].

However, toxicity restricted TTX applications. For instance, TTX could prolong local anesthesia duration of bupivacaine dexamethasone microspheres. The local anesthesia of individual bupivacaine and bupivacaine dexamethasone microspheres were 6.2 h and 31.3 h, respectively. The bupivacaine dexamethasone microspheres containing 0.05% (*w*/*w*) TTX, which lengthened the duration of local anesthesia up to 221.7 h. However, the concentration of 0.1% (*w*/*w*) TTX might lead to death of all animals. The therapeutic window of microsphere was narrow, since the lethal dose was only twice of the effective dose [26].

To improve the effect and safety of TTX, the sustained-release technique was used to prepare TTX pellets in this study. In order to evaluate the analgesia effect of TTX sustained-pellets, the writhing test was referred according to the analgesia pharmacodynamic guidelines. In addition, the acute toxicity of TTX pellets was tested in rats.

In our preliminary experiments, the LD_50_ of TTX in male and female rats with single i.v. injection was 8.54 and 10.10 μg·kg^−1^, respectively. At LD_50_, the rats appeared decreased activity, drooping spirits and decreased muscle tension. The symptom aggravated if the dosage increased to 10.53 and 11.75 μg·kg^−1^ for male and female rats, leading to the decreased rate of respiration, twitching and death. The acute intoxication symptoms indicated central nervous system depression and neuromuscular block of TTX.

Previously, the pharmacokinetic profile of the immediate-release TTX pellets was studied by our group with the dose of 100 μg/kg [16], which was well-tolerated on rat, and TTX concentrations in plasma were included in the limit of quantification. Comparing the dissolution curves of the immediate-release and sustained-release TTX pellets, 150 μg/kg was used in this study to ensure the quantification of TTX in plasma.

For i.v. injection of TTX, 1–20 μg/kg has been used for studies that focused on the cardiovascular system on rat [27], and 6 μg/kg has been well established for antinociceptive study [12]. Therefore, in our study, the dose used of TTX was set at 6 μg/kg.

The lethal dose of TTX is extremely low. Therefore, it is such a challenge to determine TTX in plasma that there are no pharmacokinetic studies of unformulated TTX via oral administration on rats reported so far. Further, TTX is unstable in the gastrointestinal tract [4], and thus the i.g. dosing of unformulated TTX would have a very low bioavailability. In addition, the bioavailability of i.g. dosing could not be improved by increasing the dose because of its strong toxicity. Therefore, it is very difficult to determine the comparative pharmacokinetic parameters of the i.g. dosing of unformulated TTX for the present sustained-release TTX pellets. Further, there are no commercially available other more TTX preparations suitable for reference so far. Therefore, data of i.v. administration was included in this paper to calculate absolute bioavailability of the sustained-release TTX pellets. Additionally, in another study that we’ve conducted [16], the immediate-release TTX pellets was orally administrated to SD rats, in which the *T*_max_ was 2 h, *t*_1/2_ was 3.23 h and the F was 6.7%. In the current study, the *T*_max_ was 5 h, *t*_1/2_ was 14.52 h and the *F* was 9.7% for the sustained-release TTX pellets (Table 5), indicating a significant improvement of TTX retention in the body.

In this paper, the enteric sustained-release TTX pellets showed excellent inhibition effect on acetic acid induced pain in writhing test in rats. The effective dose of enteric sustained-release TTX pellets were 20, 40, 60, and80 μg·kg^−1^, which was about 10 times of the effective doses of i.m. injection or s.c. injection (1, 3 or 6 μg·kg^−1^) [12,28]. Further, it is noted that the LD_50_ of the TTX pellets (840.13 μg/kg, Table 2) was also about two times as that of oral TTX (400 μg/kg, which is an estimated LD_50_ value for mammals) [29], indicating highly improved safety of the oral delivery of TTX by pelletizing.

It is worth mentioning that TTX has been tested to treat several types of cancer pain in clinical trials, in which TTX was administrated via i.m. injection. According to our data, oral administrated TTX pellets with a wider therapeutic window might be a promising formulation to treat pain in clinic. Furthermore, oral treatment greatly enhanced patient compliance. The effective dosage was also lower, which is economical for practical application.

## 5. Conclusions

In this study, the enteric sustained-release TTX pellets with greater efficacy and improved safety were prepared. The pellets were composed of sustained-release layer using Eudragit NE30D with 40% weight, barrier layer using HPMC with 5% weight and enteric layer using Eudragit L30D-55 with 20% weight, which met the enteric sustained-release preparation standard. The TTX pellets prepared in our study provided new options and evidences for pain treatment, hopefully in clinic.

## Figures and Tables

**Table 1:** Stability of tetrodotoxin (TTX) in dissolution mediums (mean ± SD, *n* = 3).

Dissolution Medium	The Content of TTX (%)		
	0 h	8 h	12 h
Pure water	100.45 ± 0.78	99.62 ± 1.04	98.46 ± 0.96
0.1 mol·L^−1^ HCl	100.39 ± 0.54	99.27 ± 0.86	97.75 ± 1.01
pH 5.8 sodium-phosphate buffer	101.59 ± 0.36	99.74 ± 0.62	101.48 ± 0.67
pH 6.8 sodium-phosphate buffer	97.51 ± 0.54	87.34 ± 1.48	84.85 ± 1.12

**Table 2:** The death by a single oral administration of sustained-release TTX pellets on Sprague–Dawley (SD) rats in acute toxicity test.

Dose (μg/kg)	♀	♂	Death
1466	5/5	5/5	10/10
1100	3/5	2/5	5/10
825	3/5	1/5	4/10
618	3/5	2/5	5/10
464	0/5	0/5	0/10
Total	14/25	10/25	24/50
LD_50_	771.65	927.98	840.13

**Table 3:** The body weights of the SD rats after a single oral administration of sustained-release TTX pellets.

Dose (μg/kg)	Gender	Body Weight (g)	
		0 d	7 d
1466	♂ ♀	241.6 ± 12.7 209.4 ± 11.08	-- --
1100	♂ ♀	233.4 ± 20.94 209 ± 11.33	235.8 ± 22.65(*n* = 3) 222.3 ± 15.17(*n* = 2)
825	♂ ♀	239.8 ± 9.28 201.6 ± 15.66	237 ± 10.82 (*n* = 4) 231.5 ± 5.12 (*n* = 2)
618	♂ ♀	242.2 ± 4.96 204.2 ± 14.35	244.5 ± 22.35 (*n* = 3) 220 ± 9.89 (*n* = 2)
464	♂ ♀	249.6 ± 3.13 202 ± 14.3	251 ± 18.06 (*n* = 5) 217 ± 17.34 (*n* = 5)

**Table 4:** Pharmacokinetic parameters of TTX in rats after i.v. TTX injection administration at 6 μg/kg body wt. (*n* = 6, mean ± SD). Reproduced from Bihong Hong et al. [16], which is licensed under a Creative Commons Attribution-(CC BY 4.0) International License.

Parameters	Unit	i.v. TTX of 6 μg/kg
AUC_0–t_	ng·h/mL	4.42 ± 0.90
AUC_0–∞_	ng·h/mL	4.63 ± 0.90
*t*_1/2_	h	0.92 ± 0.17
CL ^1^	mL/h/kg	1349.40 ± 326.75
*V*_d_^2^	mL/kg	1824.68 ± 709.84

^1^ Total body clearance; ^2^ steady state apparent volume of distribution.

**Table 5:** Pharmacokinetic parameters of TTX in rats after i.g. sustained-release TTX pellets administration at 150 μg/kg body wt. (*n* = 6, mean ± SD, median (range)).

Parameters	Unit	i.g. TTX of 150 μg/kg
*C*_max_	ng/mL	0.88 ± 0.22
*T*_max_	h	5 (3,5)
AUC_0–t_	ng·h/mL	10.77 ± 2.28
AUC_0–∞_	ng·h/mL	16.76 ± 2.93
*t*_1/2_	h	14.52 ± 2.37
CL ^1^	mL/h/kg	9156.86 ± 1447.68
*V*_d_^2^	mL/kg	192,752.13 ± 46,492.16
*F*	--	9.7%

^1^ Total body clearance; ^2^ steady state apparent volume of distribution.

## Supplementary Materials

The following is available online at https://www.mdpi.com/1999-4923/12/1/32/s1, Figure S1: Blood collection schemes of (A) intravascular (i.v.) injection of 6 μg/kg TTX and (B) intragastrically (i.g.) administration of 150 μg/kg sustained-release TTX pellets. For each group, 6 rats (260 ± 20 g), half male and half female, with jugular vein catheterization (JVC), were included. Serial blood samples were collected in heparinized tubes via the jugular vein at different time points.
