# Supplementary material for: Analgesia Effect of Enteric Sustained-Release Tetrodotoxin Pellets in the Rat
Source: Pharmaceutics. 2020 Jan 1;12(1):32. doi: 10.3390/pharmaceutics12010032 (PMC7022972; doi:10.3390/pharmaceutics12010032)
Supplement: Supplementary file 1 [file pharmaceutics-12-00032-s001.pdf]

pharmaceutics

Supplementary Materials: Analgesia Effect of Enteric Sustained-Release Tetrodotoxin Pellets in the Rat

Bihong Hong, Jianlin He, Jipeng Sun, Qingqing Le, Kaikai Bai, Yanhua Mou, Yiping Zhang, Weizhu Chen and Wenwen Huang

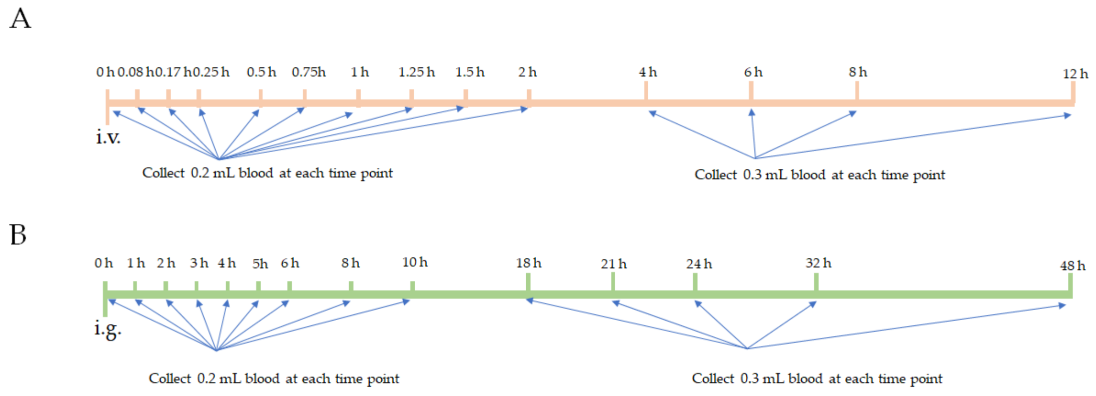

Figure S1. Blood collection schemes of (**A**) intravascular (i.v.) injection of 6 μg/kg TTX and (**B**) intragastrically (i.g.) administration of 150 μg/kg sustained-release TTX pellets. For each group, 6 rats (260 ± 20 g), half male and half female, with jugular vein catheterization (JVC), were included. Serial blood samples were collected in heparinized tubes via the jugular vein at different time points.
